# Using an electrohydraulic ankle foot orthosis to study modifications in feedforward control during locomotor adaptation to force fields applied in stance

**DOI:** 10.1186/1743-0003-6-16

**Published:** 2009-06-03

**Authors:** Martin Noel, Karine Fortin, Laurent J Bouyer

**Affiliations:** 1 Center for interdisciplinary research in rehabilitation and social integration (CIRRIS), Quebec City, Canada; 2 Department of Rehabilitation, Université Laval, Canada

## Abstract

**Background:**

Adapting to external forces during walking has been proposed as a tool to improve locomotion after central nervous system injury. However, sensorimotor integration during walking varies according to the timing in the gait cycle, suggesting that adaptation may also depend on gait phases. In this study, an ElectroHydraulic AFO (EHO) was used to apply forces specifically during mid-stance and push-off to evaluate if feedforward movement control can be adapted in these 2 gait phases.

**Methods:**

Eleven healthy subjects walked on a treadmill before (3 min), during (5 min) and after (5 min) exposure to 2 force fields applied by the EHO (mid-stance/push-off; ~10 Nm, towards dorsiflexion). To evaluate modifications in feedforward control, strides with no force field ('catch strides') were unexpectedly inserted during the force field walking period.

**Results:**

When initially exposed to a mid-stance force field (FF_20%_), subjects showed a significant increase in ankle dorsiflexion velocity. Catches applied early into the FF_20% _were similar to baseline (P > 0.99). Subjects gradually adapted by returning ankle velocity to baseline over ~50 strides. Catches applied thereafter showed decreased ankle velocity where the force field was normally applied, indicating the presence of feedforward adaptation. When initially exposed to a push-off force field (FF_50%_), plantarflexion velocity was reduced in the zone of force field application. No adaptation occurred over the 5 min exposure. Catch strides kinematics remained similar to control at all times, suggesting no feedforward adaptation. As a control, force fields *assisting *plantarflexion (-3.5 to -9.5 Nm) were applied and increased ankle plantarflexion during push-off, confirming that the lack of kinematic changes during FF_50% _catch strides were not simply due to a large ankle impedance.

**Conclusion:**

Together these results show that ankle exoskeletons such as the EHO can be used to study phase-specific adaptive control of the ankle during locomotion. Our data suggest that, for short duration exposure, a feedforward modification in torque output occurs during mid-stance but not during push-off. These findings are important for the design of novel rehabilitation methods, as they suggest that the ability to use resistive force fields for training may depend on targeted gait phases.

## Background

After disease or injury to the central nervous system, the control of locomotion is often compromised. Locomotor deficits persist even after intensive rehabilitation [1-4]. The reason for the limited success of rehabilitation is not fully understood. Original approaches are needed to improve current rehabilitation. Recent work in the field of motor learning has shown that when subjects make several reaching movements in an altered force environment ('force field'), they gradually learn to integrate the new force as part of their movement planning (modification in feedforward control; [5]). Furthermore, these modifications persist temporarily upon return to the 'normal' environment [5-8]. Such movement recalibration [9] involves modifications in muscle activation patterns [10]. These finding are of interest to the field of rehabilitation, as one could imagine designing a force field with predictable aftereffects that could have positive impacts on movement recovery [11]. Studies have now been extended to the swing phase of walking, and the application of force fields also leads to aftereffects for this more automatic movement [12-16].

However, when it comes to locomotion, care must be taken before extrapolating these interesting results to other parts of the gait cycle, due to the complex neural control and biomechanics of the walking movement. Indeed, the gait cycle can be divided into several parts, each with a functionally different contribution to movement control[17]. The present study focused on 2 of these parts: 1) 'Push-off' (~40 to 60% of movement time) is where ankle plantarflexors provide power to propel the center of mass of the body forward. Increase in power generation during this phase leads to increases in gait speed. Push-off deficits have been reported after several types of central nervous system (CNS) injury, including stroke [1,2,4] and spinal cord injury[3], leading to a reduced gait speed in these populations. 2) 'Mid-stance' (20 to ~40% of movement time), is where the body center of mass passes over the ankle. During this time, ankle plantarflexor eccentric work controls center of mass forward velocity. Mid-stance deficits are present in stroke patients (e.g. Type I patients in [18]), where premature activation of plantarflexors leads to knee hyperextension.

As part of a series aimed at better understanding the normal and pathological control of the ankle during walking, the present study evaluates if the neural control of locomotion can be modified by applying force fields specifically during either of these 2 specific phases of the walking movement. The presence of the force field will interfere with movement generation, thereby requiring a timing-specific compensation from the locomotor system.

Considering that the neural control of walking involves voluntary commands, sensory feedback and a central pattern generator (CPG; [19]), it is not obvious that the recalibrations (modification in feedforward control) reported during swing will also be present during these two portions of stance. For example, as sensory feedback plays an important role in the generation of the final muscle activation pattern, positive feedback from proprioceptors located in lower limb muscles and tendons could be used to compensate for the force field by enhancing ongoing locomotor EMG using the augmented feedback provided by the force field [20-25]. In addition, the presence of the CPG, an automatic neural control center that participates in the generation of muscle activations and that also modulates sensory input depending on where the latter arrive in the gait cycle[26], could limit the compensation for a force field depending on where it arrives in the gait cycle. Experiments applying such timing-specific force fields are therefore necessary to verify how the CNS will deal with a perturbation during stance.

Applying short duration force fields to the ankle during walking is not easy due to the dynamic characteristics of this joint. Modern high-performance robotized ankle exoskeletons now provide the means to produce such force fields. Our laboratory has recently developed a robotized ankle foot orthosis that uses a hybrid drive system (electrohydraulic) to apply forces on the ankle joint during walking [27]. This ElectroHydraulic AFO (EHO) is quite versatile in the types of forces that it can generate during walking; they include constant, elastic, and velocity dependant forces as well as force cancellation to minimize disturbance of the natural walking pattern ("backdrivability"). Furthermore, the rapid response of the machine allows switching from force production to force cancellation nearly instantaneously, thereby allowing phase-specific force fields to be produced.

In the present study, the kinematic pattern of the ankle of healthy subjects will be compared before, during, and after a 5 min exposure to force fields generated by the EHO and applied either during mid- stance or push-off. To evaluate if modification in feedforward control occurred during the exposure time, catch strides (i.e. strides without force field) will be unexpectedly inserted at several points in the force field exposure period, and ankle kinematics compared to baseline. Deviation from baseline during these catches will be interpreted as modifications in feedforward control.

## Methods

### Subjects

Experiments were performed on 12 healthy subjects (10 males and 2 females; age range 24–40 y) exempt of self-reported neurological or orthopedic disorders. All subjects gave informed consent to the protocol, which had been previously approved by the local ethics committee.

### Protocol

Subjects came to the laboratory for a single 2-hour visit. For the first 11 subjects, adaptation to two force fields (applied at 20% and 50% of gait, see below) was measured in 2 consecutive bouts of walking separated by a 5 min rest period. Order of force field presentation was randomly assigned. Each bout consisted of walking on a motorized treadmill at 1 m/s while wearing our robotized ankle foot orthosis (EHO) on the right leg. Each bout was composed of three walking periods. The first period ('control', 3 min) was used to evaluate individual baseline walking patterns. It was followed by the application of the force field ('force field', 5 min). Finally, the third walking period documented aftereffects ('post exposure', 5 min). During force field exposure, 8–10 catch strides were inserted according to a predetermined catch sequence unknown to the subjects. Catches consisted in removing the force field around strides #2, #5, #35, and on about every other 30th stride until the end of the force field exposure. Instructions to the subjects were to "*try to walk normally at all times*".

For the last subject, a control experiment was performed where a force field *assisting *plantarflexion (graded intensity) was applied during push-off. This experiment served to document the changes in ankle kinematics produced by adding 3.5–9.5 Nm of torque on top of the normal walking pattern. The subject walked on the motorized treadmill at 1 m/s while wearing the EHO on his right leg during 3 five-min walking periods. The EHO was set to force cancellation mode, and the participant was asked to walk normally. During each walking period, 7–12 cycles were inserted (pseudorandom sequence; non-consecutive strides) where a force field assisting plantarflexion was applied during push-off. This force field was essentially the reverse of FF_50% _(see below). The intensity of the force field was different in each walking period (3.5, 5.5, and 9.5 Nm).

### Force field application

**Figure 1:**
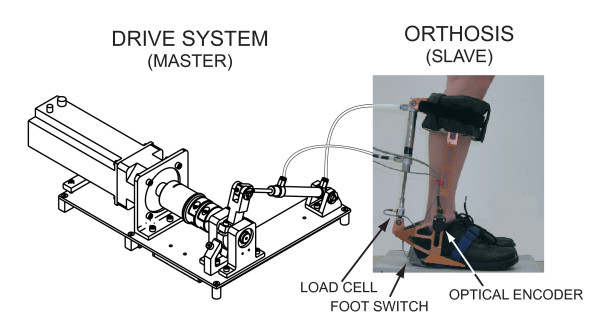
**Schematic view of the ElectroHydraulic ankle foot Orthosis (EHO)**. ***Left***. Line drawing of the drive system with an electric motor as the mechanical actuator. ***Right***. Picture of the ankle foot orthosis. These two systems are connected by pneumatic cylinders and hoses filled with water instead of air to minimize compressibility effects.

Force fields were applied to the ankle joint using a custom-designed ElectroHydraulic ankle foot orthosis (EHO; [27]). This device was optimized to operate under force control. Among its many modes of operation, the EHO provides the possibility to target specific parts of the gait cycle where to apply predetermined force fields. The EHO uses an innovative drive system combining the advantages of electric, hydraulic and pneumatic systems with light weight components (pneumatic), high power and short time constant (hydraulic), and simplified force control (electric). As shown in Figure 1, the EHO is a master-slave system composed of a drive system (electric motor) and an ankle foot orthosis. These 2 components are connected together by pneumatic cylinders and hoses that are filled with water instead of air, thereby minimizing compressibility effects. Torque control is performed in real time using a position signal from an optical encoder (US Digital Inc.) located on the joint of the orthosis, and a load cell (range +/- 220 N; Transducer Techniques Inc.) located at the extremity of the slave cylinder. The EHO is controlled in torque by a standard PID controller. In the present study, the same PID parameters were used for all subjects and both force fields. A pressure sensor located under the shoe (foot switch) was used to determine the exact moment of the impact between the heel and the ground and to calculate stride length in order to apply the force field at the appropriate time in the gait cycle. With its optimized aluminum frame, the weight of the orthosis without the shoe is 1.7 kg. Further specification can be found in Noel et al[27].

### Force field characteristics

Two force fields were used in the present study, one during mid-stance, and the other during push-off. The intensity of these perturbations was small, adjusted to provide a movement perturbation while leaving force reserve for the subjects to be able to compensate. Perturbation duration was adjusted to cover most of the phase under study, but without spreading out to other parts of the movement.

FF_20% _consisted of a parabolic torque perturbation that accelerated the ankle towards dorsiflexion during mid-stance (starting around 20% of stride). As the foot is flat on the ground during this phase of gait, FF_20% _therefore pushes the shank forward. To return ankle kinematics to normal, the subject had to resist the shank forward acceleration. FF_50% _consisted of a velocity-dependent parabolic torque perturbation that resisted ankle plantarflexion during push-off (starting around 50% of stride). To return ankle kinematics to normal, the subject had to increase plantarflexion torque during this phase of gait. During 'control', 'post exposure', and 'catch', the orthosis applied a null field (torque cancellation mode[27]), i.e. was controlled in torque with the goal of minimizing disturbances applied on the subject's ankle. This control mode actively compensates for friction and energy loss across the hydraulic circuit, thereby minimizing AFO effects on the subject's natural walking pattern. During 'force field', the robotized orthosis applied desired perturbations around 20% or 50% of stride and the null field the rest of the time.

FF_20% _was applied during 300 ms, i.e. terminated before heel off. The mathematical equation for this force field was:



where *u *= *t*/*T*, *T *= 300 *ms*, and *t *represent the onset time. *K *was set to obtain a peak torque around 10 Nm in each subject. To produce the perturbation at the right time in the gait cycle, the EHO control software used the foot switch signal to predict stride duration based on the mean of the three preceding strides.

FF_50% _was applied during 150 ms, i.e. terminated before toe off. Unlike FF_20%_, FF_50% _was dependent on ankle velocity. The reason for this difference was to make sure that the force was always applied at the same moment during push-off despite the stride-to-stride variability present in this phase of the gait cycle. The mathematical equation for this force field was:



where *A *represents the gain and *ω *the angular velocity of the orthosis. For each subject, *A *was adjusted to produce a peak torque around 10 Nm.

It must be noted that while the equations used to generate the two force fields were quite different, in both cases they produced a properly timed parabolic torque curve within the appropriate section of the gait cycle.

### Data acquisition

Relative ankle angles were recorded using the optical encoder located on the orthosis and relative knee angles were measured using an electrogoniometer (Biometrics Inc) with one end attached on the shank and the other to the thigh. Together with the foot switch and applied torque signals, they were digitized on-line by custom data acquisition software at 1000 samples/sec/channel.

### Data analysis

Using the foot switch signal, all strides were separated, synchronized on heel strike and time normalized. To determine locomotor adaptation, angular velocity of the ankle was chosen as the representative variable. Using the last 20 strides of the control as a reference ('baseline'), ankle velocity was calculated for all strides in the control, force field, catch, and post exposure conditions. Using the applied torque signal as a timing reference, mean velocity was calculated from onset of force deviation to peak force field intensity. Seven epochs were targeted for comparison:

1. baseline: mean of last 20 strides before force field application

2. force field early (initial effects): first stride in the force field

3. first catch: first null field after force field application began

4. last catch: last null field inserted during the force field application period (corresponds to stride# >200)

5. force field late: mean of last 20 strides in the force field

6. post early (initial aftereffect): first stride after force field removal

7. post late: mean of last 20 strides after force field removal

### Statistics

Considering the fact that each subject served as its own control, a one way repeated measure ANOVA was used. All conditions were tested against baseline, and compensated for repeated testing using the Bonferroni correction. Significance level was set at 0.05. It must be noted that error bars on the Figures represent the 95% confidence interval (i.e. do not include the correction for repeated comparisons) and are used simply to visually appreciate intersubject variability.

## Results

### Effects of a force field applied at 20% of stride (mid-stance)

**Figure 2:**
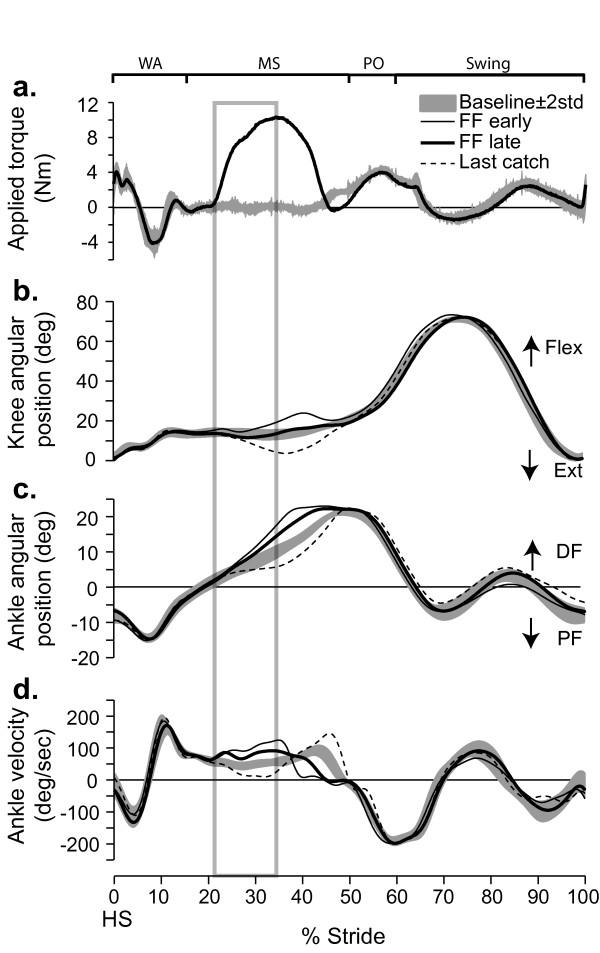
**Effects of force field 20% on joint kinematics**. **a**. Torque applied on a subject's ankle (S3) by the EHO during mid-stance. Baseline (grey band) and force field late (thick black line). Outside of the force field application zone, the EHO applied a null field to minimize its influence on the subject's walking pattern. **b**. Knee angular displacements superimposed for baseline (grey band), force field early (thin black line), force field late (thick black line), and last catch (dashed line). **c**. Ankle angular displacements superimposed for baseline (grey band), force field early (thin black line), force field late (thick black line), and last catch (dashed line). **d**. Ankle angular velocity for the same traces as in 'c'. Grey box: zone used for velocity measurement. Grey bands represent mean value ± 2 STD. For all conditions, data were synchronized on heel strike. Abbrev. *WA*: weight acceptance; *MS*: mid-stance; *PO*: push-off; *DF*: dorsiflexion; *PF*: plantar flexion; *HS*: heel strike.

Figure 2 summarizes the effects of FF_20% _exposure on knee and ankle kinematics for a representative subject (S3). Initial effects in the presence of the force field show that the ankle angular displacement deviated significantly from baseline at 25% of stride, i.e. just after force field onset ("FF early"; Figure 2C). The ankle then remained more dorsiflexed until 49% of stride i.e. the end of mid-stance. This ankle trajectory modification is partly compensated over time, as shown by a reduction in the exaggerated dorsiflexion at the end of the 5 min exposure. When the force field was unexpectedly removed (Figure 2C dashed line), the ankle now deviated from the baseline trajectory in the opposite direction, as if the subject was expecting the force to be present. As can be seen from the angular velocity trace (Figure 2D), ankle velocity increased relative to baseline in the range 22–38% of stride. Now looking at the applied torque signal (Figure 2A), this initial response corresponds very well with the period between force field onset and peak amplitude. The foot being flat on the ground during this part of the gait cycle, knee angular movements were also modified (Figure 2B). As the knee showed a behavior similar to that reported for the ankle, these data will therefore not be further discussed.

**Figure 3:**
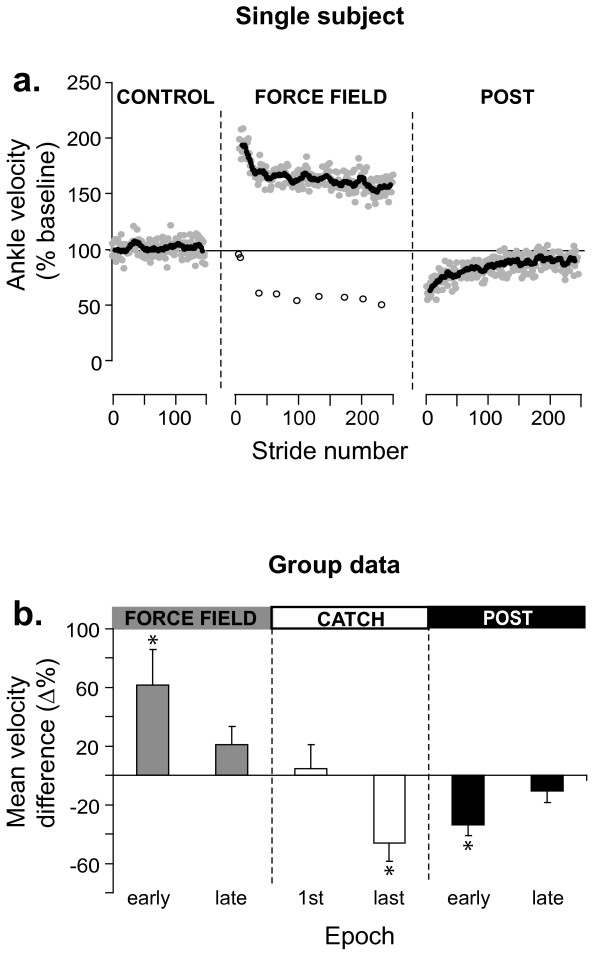
**Summary of force field 20% effects on ankle kinematics**. **a**. Time course of ankle velocity across walking conditions. Each grey symbol represents a stride. Black symbols represent an 11 points moving average. Open symbols represent catch strides. **b**. Group results (n = 11) expressed as % difference from control for the 2 epochs in each walking condition. Error bars represent 95% confidence intervals. *: Epochs statistically different from baseline (P < 0.05; repeated measure ANOVA with Bonferonni correction).

The stride-by-stride time course of ankle velocity (% of baseline) is shown for the same subject in Figure 3A. The first stride in the presence of the force field shows a large increase in ankle dorsiflexion velocity, consistent with the action of the applied torque on this joint. Velocity then gradually decreased over the first 50 strides, but did not return to baseline within the 5 min. exposure for this subject. Upon removing the force field, aftereffects consisting of a reduced ankle dorsiflexion velocity were initially observed. These effects gradually disappeared over time.

Now considering the catch strides (Figure 3A open symbols), it can be seen that the velocity of the first catch was within baseline variability. By the 3^rd ^catch (35th stride within the force field), a large reduction in ankle velocity was observed, and a plateau was then maintained.

Figure 3B presents the group results (n = 11) in the form of % difference from baseline. A value close to zero indicates that velocity was similar to baseline. A positive value indicates that velocity was larger than baseline and a negative value indicates a slowing down of the joint compared to baseline. During FF_20% _exposure, there was a significant initial velocity increase of 62% (P < 0.05). On average, this difference in velocity was compensated by the end of the 5 min. exposure (P > 0.99). Catch strides were initially not different from baseline (P > 0.99), but presented a significant difference in the direction opposite to that of force field effects by the end of the 5 min exposure (-46%; P < 0.05). Early post exposure, aftereffects were present, as shown by a 34% decrease in velocity (P < 0.05). By the end of the 5 min post-exposure, ankle velocity had returned to baseline (P > 0.99). As a complement, details regarding individual subjects' weights and peak powers produced by the EHO during FF_20% _can be found in Table 1.

**Table 1:** Subjects' weights and peak powers applied by the EHO

		FF_20%_	FF_50%_
Subject	Weight	Peak power	Peak power
	(Kg)	(Watts)	(Watts)

S1	69	8.9	- 30.0
S2	84	9.1	- 24.6
S3	56.8	16.3	- 25.6
S4	74.9	12.8	- 30.4
S5	79	5.6	- 25.6
S6	79.9	8.0	- 21.7
S7	56	7.4	- 17.1
S8	70.5	7.6	-31.0
S9	82	7.2	- 22.8
S11	72.6	10.0	- 14.3
S12	47.7	11.0	- 21.0
S13	68.2	-	15.1/24.7/45.6

Weights of our participants are presented in column 2. For FF_20% _(column 3), the sign of the peak power is positive as the orthosis applied torque in the same direction as ankle movement. For FF_50% _(column 4), peak power is negative as the orthosis applied torque in the direction opposite to the ongoing movement. In both cases however, subjects had to produce more energy to resist the force field.

### Effects of a force field applied at 50% of stride (push-off)

**Figure 4:**
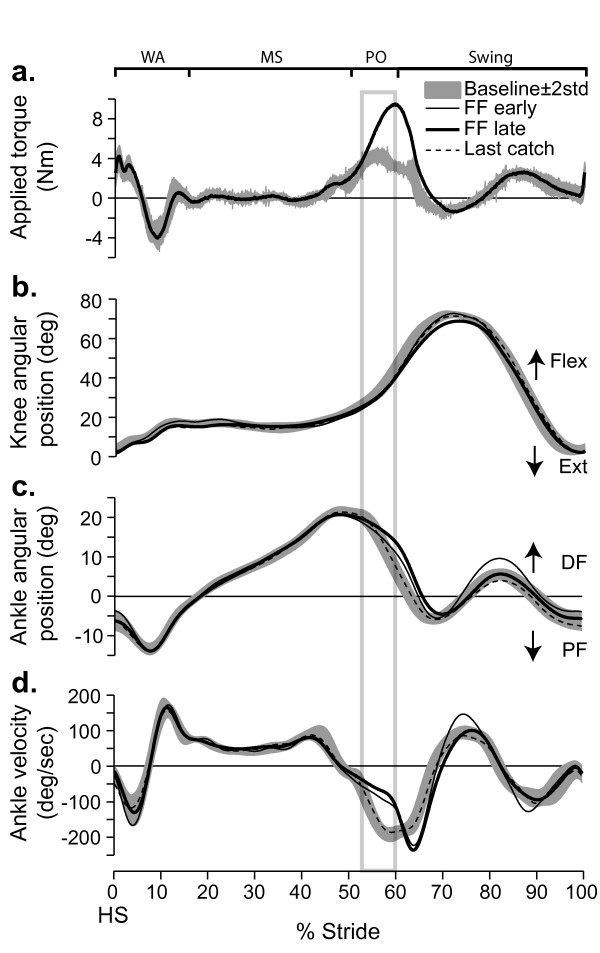
**Effects of force field 50% on joint kinematics**. **a**. Torque applied on a subject's ankle (S3) by the EHO during push-off. Baseline (grey band) and force field late (thick black line). Outside of the force field application zone, the EHO applied a null field to minimize its influence on the subject's walking pattern. **b**. Knee angular displacements superimposed for baseline (grey band), force field early (thin black line), force field late (thick black line), and last catch (dashed line). **c**. Ankle angular displacements superimposed for baseline (grey band), force field early (thin black line), force field late (thick black line), and last catch (dashed line). **d**. Ankle angular velocity for the same traces as in 'c'. Grey box: zone used for velocity measurement. Grey bands represent mean value ± 2 STD. For all conditions, data were synchronized on heel strike. Abbrev. *WA*: weight acceptance; *MS*: mid-stance; *PO*: push-off; *DF*: dorsiflexion; *PF*: plantar flexion; *HS*: heel strike.

Figure 4 summarizes the effects of a force field applied around 50% of stride for the same subject as in Figure 2. During force field exposure, ankle angular trajectories (Figure 4C) initially deviated from baseline during push-off. Figure 4D shows that the trajectory deviation was associated with a significant reduction in ankle plantarflexion velocity starting at 54% of gait. Comparing force field early and late, it can be seen that this deviation was not compensated over the 5 minute exposure. Looking at the last catch curve (Figure 4C; dashed line), it can be seen that the subject produced a trajectory similar to baseline when the force was unexpectedly removed. Comparing the knee angular displacement curves to baseline (Figure 4B), it can be seen that FF_50% _had no significant effect on knee joint kinematics.

**Figure 5:**
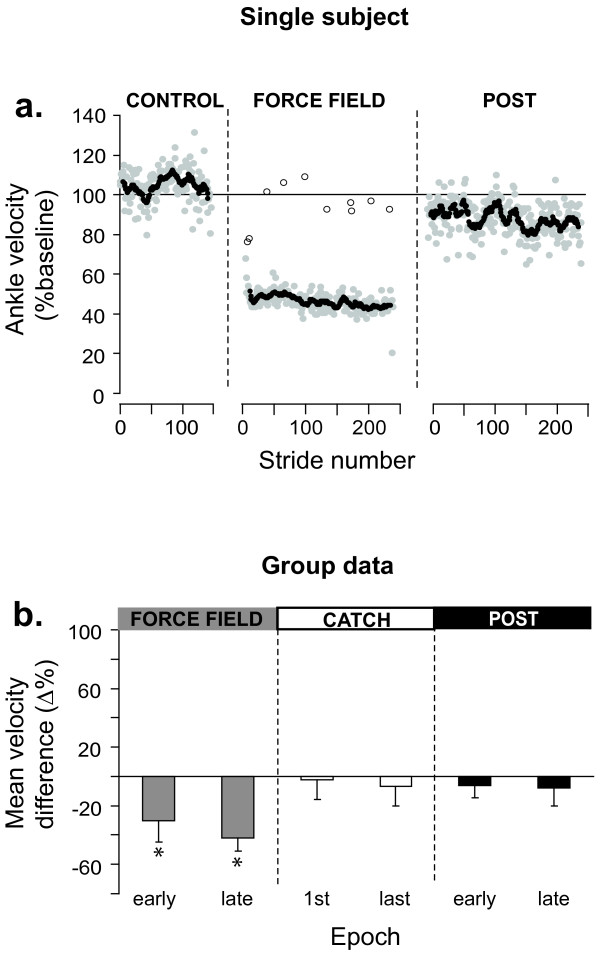
**Summary of force field 50% effects on ankle kinematics**. **a**. Time course of ankle velocity across walking conditions. Each grey symbol represents a stride. Black symbols represent an 11 points moving average. Open symbols represent catch strides. **b**. Group results (n = 11) expressed as % difference from control for the 2 epochs in each walking condition. Error bars represent 95% confidence intervals. *: Epochs statistically different from baseline (P < 0.05; repeated measure ANOVA with Bonferonni correction).

The stride-by-stride time course of ankle plantarflexion velocity (% baseline) is shown in Figure 5A for the same subject. This graph shows that there was a large immediate reduction in ankle plantarflexion velocity. FF_50%_, catch strides ankle kinematics were in the same direction as force field effects early on, and then similar to baseline. Contrary to FF_20%_, catch effects were never significantly outside of the baseline variability, in a direction opposite to force field effects. After force field removal, a residual difference in ankle plantarflexion velocity was sometimes present (e.g. Figure 5A). This difference was small, and was either a reduction (e.g. Figure 5A) or an increase (data not shown) in ankle velocity. Closer inspection of individual subject traces demonstrated that these effects were due to small changes in the exact timing of the push-off phase with respect to baseline. As all traces were synchronized on heel strike, a small difference of push-off onset time would change the velocity measured over the analyzed window, creating these residual effects. In all cases, the phase shift in push-off onset time was very small, and could not be specifically related to force field exposure.

Figure 5B summarizes the group results in the form of % difference from baseline. There was a large initial velocity reduction (-30%; P < 0.05). Velocity was still significantly different from baseline at the end of the 5 min exposure (-42%; P < 0.05). Catch strides were not significantly different from baseline (P > 0.99), and no significant aftereffects were present (P > 0.99). Details regarding individual subject's weights and peak powers produced by the EHO during FF_50% _can be found in Table 1.

### Control experiment

**Figure 6:**
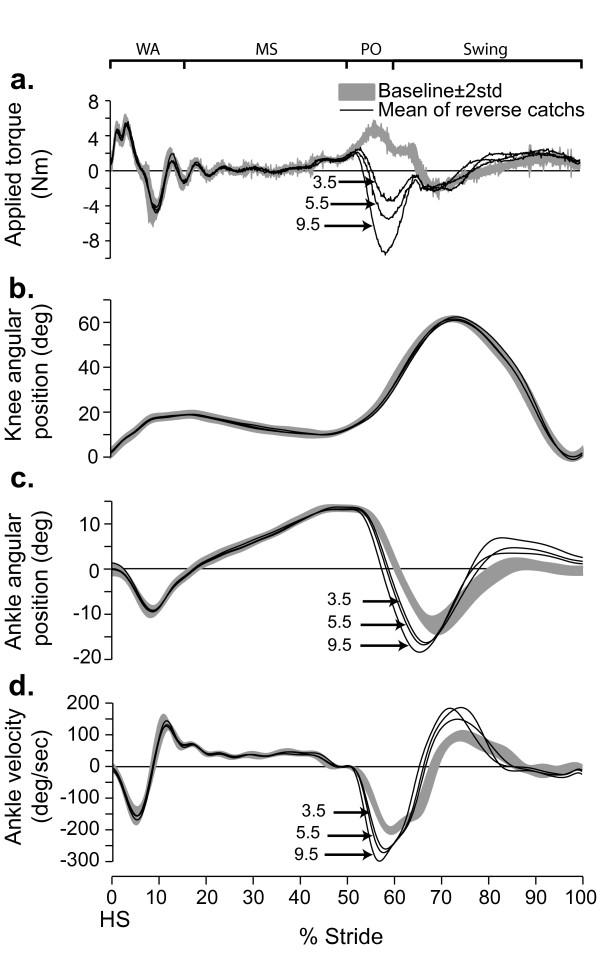
**Effects of adding torque at 50% of gait on joint kinematics**. **a**. The 3 levels of assistive torques applied on a subject's ankle (S13) by the EHO during push off (dark lines) are superimposed on Baseline (grey band); outside of the force field application zone, the EHO applied a null field to minimize its influence on the subject's walking pattern. **b**. Superimposed knee angular displacements for baseline (grey band) and the 3 levels of assistance. **c**. Superimposed ankle angular displacements for baseline (grey band) and the 3 levels of assistance. **d**. Ankle angular velocities for the same traces as in 'c'. Grey bands represent mean value ± 2 STD. For all conditions, data were synchronized on heel strike. Abbrev. *WA*: weight acceptance; *MS*: mid-stance; *PO*: push-off; *HS*: heel strike.

In a twelfth subject, the effects of *assisting *push-off with graded amounts of torque were tested. Figure 6 summarizes the results. When the force field was unexpectedly applied (see Methods), ankle plantarflexion was larger (Figure 6C) and ankle velocity increased (Figure 6D). The magnitude of the effects was proportional to torque intensity (Figure 6A), but even at the smallest torque intensity tested (-3.5 Nm) large changes in ankle velocity were observed. Similar to FF_50%_, knee kinematics were not modified by force field application (Figure 6B).

## Discussion

### Five minute exposure to FF_20% _induces a rapid modification in feedforward control during mid-stance

When exposed to a force field during mid-stance (FF_20%_), subjects exhibited an initial error in lower limb kinematics (increased dorsiflexion velocity). This error was gradually compensated in about 50 strides. This rapid time course of adaptation is within the range of other studies where subjects walked with force fields applied during the swing phase of gait [12-16].

During force field exposure, 8 to 10 catch strides (i.e. gait cycles with a null field) were inserted at moments unexpected by the subjects. On the first catch, inserted in the initial force field exposure period where the kinematic error was largest, dorsiflexion velocity was similar to baseline. On the catches inserted after adaptation occurred, catch stride dorsiflexion velocity deviated from baseline in the direction opposite to force field effects. The facts that later catches were different from baseline, and in opposite direction with force field-induced kinematic error suggest that modifications in feedforward control occurred as part of the process of adaptation to FF_20% _[8]. Furthermore, the fact that catch effects developed progressively is compatible with gradual movement recalibration [9] possibly through an iterative updating of an internal model of limb dynamics [5-7]. Looking at the post exposure period, aftereffects were present, and gradually disappeared over time. This again corroborates results obtained for force fields applied during swing in previous studies [12-16] and supports the notion that the recalibration was a robust process, requiring several strides before the normal motor pattern returned.

### Five minute exposure to FF_50% _does not modify feedforward control during push-off

When exposed to a resistive force field during push-off, subjects initially showed a large reduction in ankle plantarflexion velocity. With repeated exposure to FF_50% _(> 200 strides), subjects did not compensate by increasing velocity over this zone of the gait cycle. Catch strides presented kinematics similar to baseline regardless if they were inserted early or late in the FF_50% _exposure period. Finally no aftereffects were observed. Together, these results suggest that there were no modification in feedforward control during FF_50% _exposure.

The striking element regarding FF_50% _is that a force field with a relatively small intensity (~10 Nm) applied during push-off produced a significant and persistent reduction in plantarflexion velocity. This finding may at first glance look surprising considering that the neural control of locomotion is capable of important torque/power modulation during this phase of the movement to accommodate for changes in walking speed. Comparing ankle torques and powers during push-off at slow, moderate and high walking speeds [17], it can be seen that torque and power reserve are available at this phase of gait, and therefore that the lack of adaptation did not result from a biomechanical limit of the locomotor system.

While catch strides are a simple and powerful tool to study modifications in feedforward control, they also have limitations. In the present study, one of these limitations is that while the presence of kinematic difference between a catch stride and control is a direct proof of a modification in feedforward control, an absence of difference could either be due to a lack of change in feedforward control or to a mechanical situation where the modified muscle activation pattern is not sufficiently different from baseline to overcome the mechanical impedance of the system at the given phase of the walking movement. To address this limit of the method, an additional experiment was performed where the EHO was used to *assist *the ankle movement during push-off. Here again, movement kinematics were compared to baseline. Figure 6 clearly shows that assisting push-off with as little as 3.5 Nm was more than sufficient to produce a significant increase in ankle plantarflexion velocity. As this force field intensity is only about one half of what was required to compensate for FF_50%_, this experiment therefore rules out the possibility that the lack of catch effects during FF_50% _exposure were due to a large mechanical impedance around the ankle during push-off, and supports the interpretation that the subjects did not modify their feedforward control over the 5 min exposure.

Several possibilities can be proposed to account for the lack of adaptation and modifications in feedforward control during FF_50% _exposure. One is that *exposure duration *was not long enough for compensatory mechanisms to start acting during this phase of the gait cycle. In our group of participants, a 5 min. exposure represented between 222 and 290 strides. While this number is much larger than the number of strides required to adapt to force fields applied to the swing phase of walking (range 4–124; [12-14]), the work of Gordon and Ferris[28] showed that 24 min. of exposure were required on average to adapt to an assistive force applied during push-off. However, contrary to the present study, a modification from their initial effect in the force field was already visible after 1 minute of exposure (their Figure 4). Another possibility is that *force field duration *was too short. However, force field duration was long enough to induce a large kinematic error (ankle velocity reduction of 30 to 42% of baseline; Figure 3B), and therefore presumably sufficient to activate sensory receptors to signal the movement error to the CNS circuitry. A third possibility is that participants could have been relying on feedback mechanisms to compensate for the force field. Considering that positive force feedback is available during walking [20-25], having a resistive force field applied on the ankle would increase muscle loading, and through positive feedback loops, force output could be increased. However, contrary to feedforward control, feedback compensation would arrive delayed with respect to force field onset. Furthermore, catch strides would be similar to baseline as the augmented sensory feedback would not be there to trigger the assistive reflex pathways. Contrary to the gradual update of an internal model that requires several iterations before reaching a steady-state, the use of augmented feedback would reach steady-state as early as on the first stride, as the feedback loop would be activated after force field onset. As measuring feedback contribution to adaptation was not the focus of this study, such mechanisms could not be directly measured here due to the gradual onset and offset of the force field (parabolic shape) and the absence of electromyographic (EMG) recordings. Such measurements represent an interesting future direction.

Nevertheless, the fact that *the same *participants adapted to FF_20% _but not to FF_50% _clearly demonstrates that the adaptive control mechanisms involved in force field adaptation are not the same for these 2 parts of the stance phase.

### Using exoskeletons to unravel neurophysiological mechanisms underlying ankle control during stance

It was clearly shown in this study that inserting catch strides during force field exposure provided a valuable tool to study the feedforward contribution to locomotor adaptation during stance. In addition, imposing phase-specific force fields allowed separating different types of sensorimotor integrations across the gait cycle. Ankle exoskeletons such as the EHO are essential to the realization of such experiments and open to a completely new way of addressing complex neurophysiological questions about the neural control of normal and later pathological human locomotion. The present study is only one example of how the EHO characteristics can be exploited; its simple force control, small time constant, large range of motion, and light weight[27], are available for additional experimental designs. Furthermore, the fact that the actuator located on the EHO is a cylinder filled with water makes the system very low in electromagnetic interference. Further studies will therefore have the possibility to add EMG recordings to data collection, and address the motor strategies (e.g. muscle groups involved, muscle activation patterns, etc) associated with the reported kinematic modifications. Combined with other methods such as reflex testing, the EHO could even be used to investigate the neural pathways underlying adaptation/compensation.

## Conclusion

Taken together, these results suggest that there is a difference in the way the CNS deals with force fields applied at the ankle during mid-stance and push-off.

FF_20% _showed similar results to studies applying force fields during swing; FF_50% _did not. Unfortunately, to our knowledge there is no equivalent study to compare FF_50% _to. For push-off, a lack of feedforward modification may have important implications for rehabilitation training based on aftereffects [11], as aftereffects are really just another manifestation of modified feedforward control. Alternative approaches must be considered to provide additional insight into the control of push-off. For example, Gordon and Ferris [28] perturbed the relationship between ankle muscle coordination and ankle joint dynamics using a different ankle exoskeleton that assists plantarflexion based on plantarflexor EMG signals. They showed a gradual motor adaptation during push-off and retention of this learning at 72 hrs. Such results suggest that higher level approaches may be necessary to improve push-off control after CNS injury. Finally, while our study looked at feedforward modifications to forces applied *within *mid-stance and push-off, it is possible that force fields applied just *prior to *these phases could also influence movement control of these critical moments in the gait cycle. Additional experiments are required to address this point.

## Competing interests

The authors declare that they have no competing interests.

## Authors' contributions

MN participated in the design of the study, was responsible for the software modifications and control of the robotized orthosis, participated in data collection/analysis, and helped to draft the manuscript. KF was responsible for data collection, carried out the data analysis, and performed the statistical analysis. LJB conceived the study, participated in its design and coordination, and drafted the manuscript. All authors read and approved the final manuscript.
